# Improving the Size Homogeneity of Multicore Superparamagnetic Iron Oxide Nanoparticles

**DOI:** 10.3390/ijms21103476

**Published:** 2020-05-14

**Authors:** Barry J. Yeh, Tareq Anani, Allan E. David

**Affiliations:** Department of Chemical Engineering, Samuel Ginn College of Engineering, Auburn University, Auburn, AL 36849, USA; bjy0001@auburn.edu (B.J.Y.); tba0008@auburn.edu (T.A.)

**Keywords:** magnetic nanoparticle, SPION, size distribution, magnetic attraction, diffusion, magnetic field flow fractionation (MFFF)

## Abstract

Superparamagnetic iron oxide nanoparticles (SPIONs) have been widely explored for use in many biomedical applications. Methods for synthesis of magnetic nanoparticle (MNP), however, typically yield multicore structures with broad size distribution, resulting in suboptimal and variable performance in vivo. In this study, a new method for sorting SPIONs by size, labeled diffusive magnetic fractionation (DMF), is introduced as an improvement over conventional magnetic field flow fractionation (MFFF). Unlike MFFF, which uses a constant magnetic field to capture particles, DMF utilizes a pulsed magnetic field approach that exploits size-dependent differences in the diffusivity and magnetic attractive force of SPIONs to yield more homogenous particle size distributions. To compare both methods, multicore SPIONs with a broad size distribution (polydispersity index (PdI) = 0.24 ± 0.05) were fractionated into nine different-sized SPION subpopulations, and the PdI values were compared. DMF provided significantly improved size separation compared to MFFF, with eight out of the nine fractionations having significantly lower PdI values (*p* value < 0.01). Additionally, the DMF method showed a high particle recovery (>95%), excellent reproducibility, and the potential for scale-up. Mathematical models were developed to enable optimization, and experimental results confirmed model predictions (*R*^2^ = 0.98).

## 1. Introduction

Magnetic nanoparticles (MNPs) are an important class of nanomaterials that have been used in several biomedical applications including hyperthermia [1], magnetic resonance imaging (MRI) [2], drug and gene delivery [3,4], in vivo cell tracking [5], and tissue engineering [6]. Some methods of MNP synthesis yield particles with multicore structures, comprised of a cluster of individual crystals, as opposed to single-core particles, bound together by a protective matrix that inhibits further changes to the number of cores per particle [7]. Examples of polymers used for coatings include polysaccharides (e.g., Dextran and carboxydextran), such as for encapsulating the 4–8 nm magnetic cores of the MRI contrast agents Endorem^®^ (hydrodynamic diameter of 80–150 nm) [8] and Resovist^®^ (hydrodynamic diameter of 45–60 nm) [9]; molecular coatings (e.g., citric acid and dimercaptosuccinic acid) [10]; and silica coatings [11]. The number, size, and spatial distribution of the cores and their magnetic interactions directly impact the observed physiochemical and magnetic properties of the particles [12]. For example, it has been shown that the effectiveness of MNPs used in magnetic hyperthermia is directly related to the inter-core interactions [12]. Control over these properties, however, can be challenging, with small changes to reaction conditions (e.g., temperature, concentrations of reagents and surfactants, energy used, and reaction time) resulting in large variation in physical properties and heterogeneity, with various shapes and broad size distributions [12,13,14].

It is well known that the size and size distribution of nanoparticles influence its cargo-loading efficiency, pharmacokinetics, biodistribution, tumor-targeting properties, kinetics of cellular internalization, and toxicity [15,16,17]. The efficiency of MNPs in MRI, hyperthermia, and drug delivery, therefore, is a function of the distribution of physiochemical properties of the particles [13,18,19,20,21]. For example, medium-sized nanoparticles (10–100 nm) are considered pharmacokinetically optimal for tumor-targeting applications as they minimize clearance by the mononuclear phagocyte system while also avoid renal clearance and offer the potential for tumor accumulation [22,23,24]. However, even within the 10–100 nm range, small changes in particle size have a significant effect on the pharmacokinetic behavior and the permeation of particles through tumors [24]. A broad size distribution consisting of small, medium, and large nanoparticles results in multistage clearance, heterogeneous tumor accumulation, and nonuniform MRI contrast [25,26,27]. For instances when synthesis of homogenous MNPs is challenging, an alternative approach to reduce polydispersity is to isolate specific fractions of particles from a mixture.

A number of post-synthesis techniques, including filtration, size exclusion chromatography (SEC), differential centrifugation, and density gradient centrifugation, have been employed to separate subpopulations of nanoparticles by size and to generate more homogenous fractions. These methods, however, suffer from poor control and scalability [28,29,30,31]. Filtration and SEC techniques separate nanoparticles based on differences in hydrodynamic volume of the particles and by varying particle mobility through a porous filter or gel [32]. In one instance, Briley-Saebo et al. used vacuum filtration to isolate a fraction of smaller particles (ø 15.1 nm, polydispersity index (PdI) = 0.2) from a broader sample of the MRI contrast agent Feridex (ø 121.2 nm, PdI = 0.4) [14]. They demonstrated that the pharmacokinetics and uptake of MNPs by intraplaque macrophages associated with atherosclerosis differ significantly between fractionated and non-fractionated Feridex. However, filtration-based separations are limited by the homogeneity of pore sizes of the filter membrane [33]. SEC, on the other hand, typically requires the addition of a surfactant that can destabilize magnetic fluids, and the technique is limited in separating larger MNPs due to appropriateness of SEC columns [28].

In differential centrifugation, particles are separated based on size and density by varying the centrifugation speeds. In this method, a mixture of heterogeneous particles is centrifuged and the pellets are collected, and the process is repeated at increased centrifugal speeds to collect subsequent fractions [29]. It is, however, difficult to achieve complete removal of the supernatant without disrupting the pellet, and as the particles of a given size are distributed throughout the medium and must travel varying distances to reach the forming pellet, this technique also results in a polydisperse sample [34].

In another method, magnetic fields can be used to separate MNPs based on their size, material composition, and magnetic susceptibilities [34]. Magnetic field flow fractionation (MFFF) utilizes an external magnetic field acting perpendicular to hydrodynamic forces acting on particles in a flow stream. Typically, MNPs are first immobilized on the surface of magnetic beads by an applied magnetic field. Introduction of a fluid flow and gradual decrease of the magnetic field strength allows for the elution of layers of particles from the bead surface, which are collected. However, since the initial sedimentation of MNP results in a distribution of sizes in each layer due to variable proximity of the MNP to the bead surface, the recovered samples are still heterogenous. While MFFF is faster and has a higher capacity compared to SEC, there is still much room for improvement to obtain homogenous samples [28]. For example, a comparison of ultracentifugation, ultrafiltration, and magnetic separation showed that the sample size distribution remained polydispersed [35].

To improve the quality of MNP separation via magnetic fields, we here introduce a new technique labeled diffusive magnetic fractionation (DMF). We hypothesized that a process that uses a pulsed magnetic field (PMF), which exploits size-dependent difference in the magnetic forces exerted on particles and size-dependent diffusivities of particles, will produce a more homogenous layering of MNP around the magnetic beads compared to the use of a constant magnetic field. Subsequent release and recovery of layers of particles would yield a more homogenous sample compared to what is achievable with traditional MFFF methods. Here, we demonstrate that DMF provides significantly improved performance over the conventional MFFF method in separating a heterogeneous population of multicore MNPs into homogenous fractions with distinctive sizes. Superparamagnetic iron oxide nanoparticles (SPIONs) were chosen as the model MNP because of their relatively safe toxicity profile and well-established history as a clinically approved MRI contrast agent [36,37,38].

## 2. Results

### 2.1. Fractionation of Starch-Coated SPIONs with DMF versus MFFF

In this study, we introduce the DMF method as a potential improvement over conventional MFFF for the fractionation of SPIONs from a mixture having a broad size distribution. Starch-coated SPIONs (ø 105 ± 1.7 nm; PdI = 0.24 ± 0.01) were separated into nine size fractions using three different techniques: (1) MFFF, in which particles suspended in a flowing fluid are magnetically captured from the flow field; (2) DMF-0 (with no PMF cycles), which differs from MFFF in that particles are magnetically captured from a stagnant fluid body; and (3) DMF-9 (with nine PMF cycles), where particles are alternatively magnetically captured from a stagnant fluid and are allowed to diffuse into the stagnant fluid.

**Figure 1:**
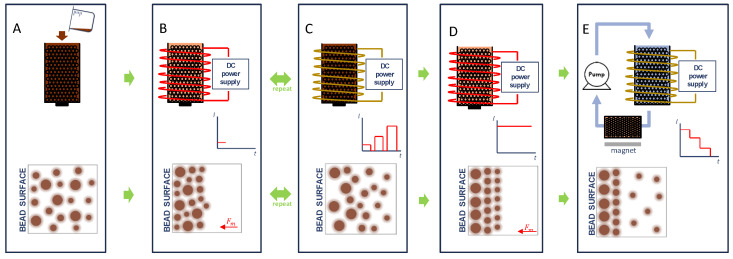
Illustration of the Diffusive Magnetic Fractionation (DMF) process for sorting of magnetic nanoparticles: (**A**) Superparamagnetic iron oxide nanoparticles (SPIONs) are introduced to a column filled with magnetizable beads. (**B**) A low magnetic field (0.4 mT) is applied, and SPIONs are allowed to accumulate on the bead surface. (**C**) The magnetic field is then removed, and SPIONs then diffuse away from bead surface due to Brownian motion at a rate inversely proportional to particle size. The ON and OFF cycle is repeated (9 total pulses for DMF-9) with an increasing applied magnetic field from the initial to final pulse. (**D**) During the final cycle, a maximum magnetic field (24 mT) was maintained for an extended period of time to capture all particles. Finally, (**E**) captured particles are then fractionated, layer-by-layer, by applying a fluid flow field and by gradually decreasing the applied magnetic field strength. The released SPION fractionations are captured by a second column that is filled with magnetizable beads and is exposed to a 220-mT bar magnet.

As shown in Figure 1A, the DMF method begins with the introduction of SPIONs to a column filled with magnetizable beads. Subsequent magnetization of the beads (Figure 1B) leads to accumulation of the SPIONs on the bead surface. While larger particles experience a greater acceleration due to the applied magnetic field, the random distribution of particles of different sizes at varying distances from the bead surface (i.e., SPIONs of a particular size found both near and far from the bead surface) leads to a heterogeneous stacking of particles. When the magnetic field is removed, as shown in Figure 1C, the SPIONs can then diffuse away from the bead surface due to Brownian motion at a rate inversely proportional to particle size as described by the Stokes–Einstein equation [31]. Smaller particles would, therefore, be expected to on average diffuse further away from the bead surface over a finite time period. When the magnetic field is again applied, the predominance of larger particles near the surface and smaller particles away from the surface can be expected to yield a stratified layering on the bead surface according to particle size. We hypothesized that the repeated capture and release of particles would eventually lead to the layering of SPIONs on the bead surface with each layer containing a more homogenous size distribution than that of the sample as a whole, as shown in Figure 1D. In the case of DMF-9, the strength of the applied magnetic field was increased from an initially weak magnetic field (0.4 mT), which will presumably most effectively attract the larger SPIONs and possibly smaller SPIONs closer to the magnetic bead surface, to a maximum field of 24 mT during the last PMF cycle. This final magnetic field, selected based on limitations of available equipment, was maintained for an extended period of time to ensure complete immobilization of all SPIONs in the sample. Finally, as shown in Figure 1E, the captured particles are then fractionated, layer-by-layer, by applying a fluid flow field and by gradually decreasing the applied magnetic field strength. It should be noted that the expected arrangement of SPIONs on the bead surface following DMF-9 is from large to small and that the particles would be recovered from small to large by decreasing the applied magnetic field. The effectiveness of separation of the three methods was determined by comparing the sample polydispersity index (PdI), a dimensionless number that represents the nonuniformity of the hydrodynamic size distribution with smaller numbers indicating a more homogenous sample [26].

**Figure 2:**
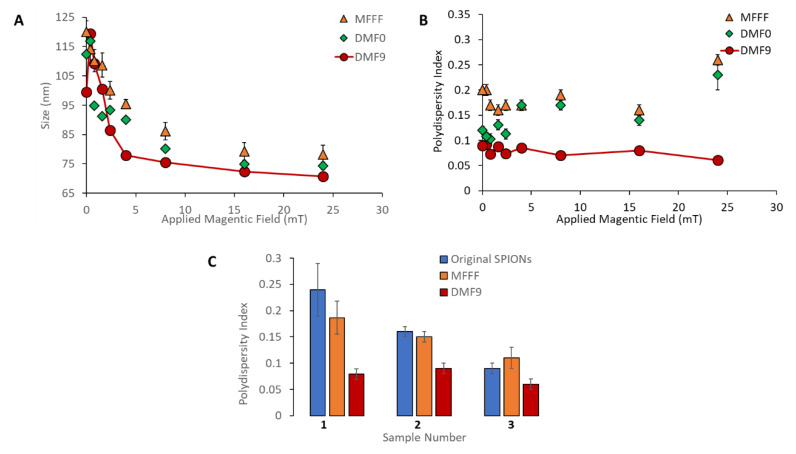
Comparison of the magnetic field flow fractionation (MFFF), DMF-0, and DMF-9 methods for fractionation of a heterogeneous population of multicore, starch-coated SPIONs (ø 105 ± 1.7 nm; polydispersity index (PdI) = 0.24 ± 0.01) into 9 different subpopulations. (**A**) The Z-average hydrodynamic diameter and (**B**) PdI values are shown for all 9 subpopulations gathered at different applied magnetic fields. Compared to samples obtained by MFFF and DMF-0, all nine subpopulations produced by DMF-9 had significantly improved PdIs (*p*-value < 0.05). (**C**) Three different batches of ‘original’ starch-coated SPIONs with different PdI values (0.24 ± 0.01, 0.16 ± 0.01, and 0.09 ± 0.01) were fractionated by the MFFF and DMF-9 methods, and the average PdI values of the 9 fractions are shown.

As seen in Figure 2A, all three separation methods were able to take an initial sample with broad size distribution (PdI = 0.24 ± 0.05) and produce subpopulations of SPIONs with average diameters ranging from 70–120 nm under the conditions tested. While the range of particle sizes recovered was similar, a higher batch-to-batch variation in average size of particles was observed with MFFF compared to DMF-0 and DMF-9, as determined by the standard deviation of mean particle size. Additionally, when the PdIs shown in Figure 2B are considered, it is clear that DMF-9 yielded the most homogenous samples, with an average PdI of 0.079 ± 0.01 across the nine fractions, followed by DMF-0 (average PdI = 0.14 ± 0.04) and MFFF (average PdI = 0.19 ± 0.03). All nine fractions obtained using MFFF had PdI > 0.15. It is interesting to observe that, while DMF-0 produced SPION fractions with significantly improved PdIs compared to MFFF (*p*-value < 0.05) at lower magnetic fields (≤2.4 mT or for larger particles), there was no significant improvement in the PdI values for the fractions of smaller SPIONs released at higher magnetic fields (>2.4 mT).

Compared to samples obtained by MFFF and DMF-0, all nine subpopulations produced by DMF-9 had significantly improved PdIs (*p*-value < 0.05), thus validating the approach of combining magnetic attraction with diffusive dispersion to create a more organized multilayer structure of particles around the beads prior to their subsequent recovery. The higher PdI values obtained with MFFF and DMF-0 indicate the presence of heterogeneous layers, with some fraction of the larger particles residing in the outer layers (farthest from the magnetic beads) and smaller particles residing in the inner layers (closer to the magnetic beads). In addition, the average size of the smallest fractionations (i.e., at higher magnetic field strengths) is consistently smaller in DMF-9 compared to MFFF and DMF-0, indicating the likely presence of larger sized particles mixed with smaller particles in fractionations produced by those methods and demonstrating the superiority of DMF-9 for capture and isolation of the smallest particles. Finally, it should also be noted that the standard deviation of the measured PdIs (*n* = 3) is significantly smaller for DMF-9 than for MFFF and DMF-0, indicating greater robustness of the DMF process for yielding reproducible results.

To further test the reproducibility of the DMF process, three different batches of starch-coated SPIONs were obtained from a commercial vendor (Chemicell GmbH, Berlin, Germany) with starting PdI values of 0.24 ± 0.01, 0.16 ± 0.01, and 0.09 ± 0.01, and each batch was separated by the MFFF and DMF-9 methods. As shown in Figure 2C, the DMF-9 method yielded SPIONs with significantly smaller PdIs compared to the MFFF method (*p* < 0.05) for all three batches of SPIONs. It is interesting to note that, in sample #3, the cumulative PdI of all MFFF fractions was calculated to be greater than that of the original, unfractionated SPION. This appears to be the result of high variability in the MFFF performance, as observed by the range of PdIs across MFFF particle fractions seen in Figure 2B, especially with small particles released at the higher magnetic fields. Since the PdI is proportional to the ratio of distribution width-to-average particle size, such an outcome could be expected if the size but not the distribution width is decreased. In all instances studied in this work, the DMF method was found to provide consistent performance with low variations in measured PdIs.

### 2.2. Versatility and Scalability of the DMF Method

**Figure 3:**
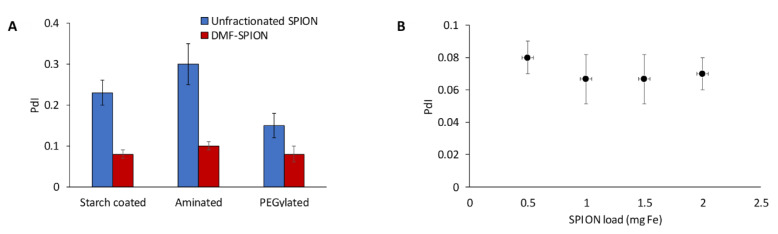
(**A**) Starch-coated, aminated, and PEGylated SPIONs having initial PdI values of 0.23 ± 0.03, 0.3 ± 0.05, and 0.15 ± 0.03, respectively, were fractionated with DMF to yield average PdI values across all samples of 0.08 ± 0.01, 0.1 ± 0.01, and 0.08 ± 0.01, respectively. (**B**) Scale-up of the DMF process with 100 nm starch-coated SPIONs did not significantly affect performance as low PdIs were maintained.

The ability of DMF-9 to fractionate polydisperse SPIONs with different surface coatings was also investigated. First, starch-coated SPIONs were surface functionalized with amine groups and then modified with 5 kDa MW polyethylene glycol (PEG). DMF-9 was then used to, separately, fractionate the starch-coated, aminated, and PEGylated SPIONs, and the particle size distribution of obtained samples was measured. As shown in Figure 3A, PdI values of DMF-9 samples were significantly improved compared to the corresponding, unfractionated SPIONs. It is interesting to note that the minimum magnetic field strength necessary to fully immobilize 1 mg of each SPION varied according to the material (12, 24, and 34 mT for starch-coated, aminated, and PEGylated SPIONs, respectively). This was likely due to differences in the various forces governing interparticle interactions. For example, aminated SPIONs can be expected to experience strong electrostatic repulsion due to high surface charge (zeta potential = +45 ± 2 mV), while the bulky PEG polymer induces steric repulsion between PEGylated particles. Since particles must sediment on each other for capture, any increase in repulsive forces between particles would need to be overcome by magnetic forces.

In addition to the significant improvement in separation quality, DMF-9 retains many of the advantages offered by MFFF, including its high particle recovery and excellent potential for scalability. The recovery yield of DMF was determined by measuring iron concentrations in SPION suspensions before and after DMF separation. Specifically, 0.4 mg Fe of multicore, starch-coated SPIONs (intensity-weighted hydrodynamic diameter = 105 ± 1.7 nm) was fractionated by the DMF process into eight subpopulations of varying size. The total SPION recovery in all samples was 0.38 mg Fe, which is equivalent to 95% particle recovery.

To assess the scalability of DMF, four magnetic columns of different sizes (0.25, 0.5, 0.75, and 1 mL) were filled with increasing SPION loadings of 0.5, 1, 1.5, and 2 mg SPIONs, respectively. Averaging PdIs of all samples obtained from a given column showed no significant difference between the four magnetic columns, with average PdIs of 0.08, 0.07, 0.07, and 0.07, respectively (see Figure 3B). Additional large-scale experiments are needed, but these results show that DMF could potentially be scaled-up without compromising its performance.

### 2.3. Mathematical Modeling of DMF

#### 2.3.1. Force Balance on an MNP under the Influence of Flow and Magnetic Fields

In an effort to optimize DMF performance, a mathematical model was developed to further study the mechanism by which SPIONs are fractionated. The behavior of MNPs in the presence of an external magnetic field is a function of several factors, including magnetic and drag forces, Brownian motion, and van der Waals forces [39]. Among these forces, it was assumed that the magnetic and drag forces dominate the behavior of the SPIONs when under the influence of a magnetic field gradient. The attractive magnetic force (*F_m_*) experienced by a single SPION in the vicinity of a magnetized bead is expressed as

(1) $$F_{m}=\nabla \left(m\cdot B\right)=\nabla \left(\frac{4}{3}\pi R_{np}^{3}\rho \chi \vec{H}\cdot \mu \vec{H}\right)$$

where *m* is the magnetic moment of the nanoparticle; B is the induced magnetic field generated by a single iron bead; $R_{np}$ is the radius of the magnetic nanoparticle; *ρ* is the density of SPIONs; χ is the magnetic susceptibility; *µ* is the magnetic permeability; and $\vec{H}$ is the magnetic field surrounding a magnetized, spherical bead, as calculated using a Legendre function [40].

On the other hand, the drag force (*F_d_*) exerted on a small, spherical nanoparticle by a viscous fluid at low Reynolds number (Re < 20) is calculated using the Stokes formula [31,41], defined as

(2) $$F_{d}=6\pi \eta R_{np}v$$

where η is the fluid viscosity, $R_{np}$ is the particle hydrodynamic radius, and v is the fluid velocity. With the magnetic force proportional to $R_{np}^{3}$ and the drag force proportional to $R_{np}$, the size-dependent balance between magnetic and drag forces on the particles can be exploited to optimize their separation. In general, particles are captured on the magnetized bead surface when $F_{m}>F_{d}$ and are released into the surrounding fluid when $F_{m}<F_{d}$.

For simple analysis, the multicore SPIONs were approximated as a particle having a single magnetic volume and the magnetic moment was determined by the product of this volume and the magnetic saturation (i.e., a single particle with an effective magnetic dipole). This simplification disregards the complex structure found with multicore particles and the dynamics of their interaction, which have been shown to be important for other processes [42,43]. However, as our model is registered to experimental data, the obtained fitting parameters (e.g., effective diffusivity of SPIONs in water) likely encapsulate some of these effects. In addition, our analysis assumed a negligible difference in the particle hydrodynamic size and magnetic core size, allowing for the same radius, $R_{np}$, to be used for magnetic and drag force calculations. While a more detailed study of these properties may lead to additional insight of the DMF method, the approximations used are sufficient to capture the important trends for MNP separation, as shown below.

#### 2.3.2. Simulation of SPION Attraction by an Applied Magnetic Field

The mass transport of SPIONs within the magnetic column is described by the equation of continuity:

(3) $$\frac{\partial c}{\partial t}+\nabla \cdot \vec{J}=0$$

where the particle concentration, *c*, is a function of time, *t*, and position, *x* and where the total flux, $\vec{J}$, is a summation of the contributions from SPION diffusion, $\vec{J_{D}}$, and the net effect of the magnetic and drag forces, $\vec{J_{F}}$, where $\vec{J_{F}}\neq 0$ when a magnetic field is applied (ON phase of the DMF) and $\vec{J_{F}}=0$ during the OFF phase of the DMF. During the ON phase of DMF, the equation of continuity can be rewritten as Equation (4).

(4) $$\frac{\partial c}{\partial t}=D\nabla^{2}c-\nabla \cdot \left(\vec{v}c\right)$$

where D is the diffusivity and $\vec{v}$ is the particle drift velocity. The time-dependent solution to this equation, which is later confirmed experimentally, takes the form of Equation (5).

(5) $$c=c_{0}e^{-\beta t_{m}}$$

where $t_{m}$ is the duration of applied magnetic field (i.e., the pulse width of the PMF or the time duration for the ON cycle) and the fitting constant β is a function of particle size, $R_{p}$, and position, *x*. Using a steady-state, position-dependent solution for Equation (4) and $r_{\left(x\right)}$ obtained from literature [44], the parameter β is expressed as follows:

(6) $$\beta =-Dr_{\left(x\right)}^{\prime \prime }+\vec{v}r_{\left(x\right)}^{\prime }+\vec{v^{\prime }}r_{\left(x\right)}$$

The particle drift velocity can be obtained from the net force acting on a single SPION, which is assumed to be equal to the sum of the magnetic attraction force, $F_{M}$, and Stokes’ drag force, $F_{D}$:

(7) $$F=F_{M}-F_{D}=\nabla \left(\vec{m}\cdot \vec{B}\right)-6\mu \eta R_{p}\vec{v}$$

where $\vec{B}$ is the applied magnetic field; η is the dynamic viscosity; $R_{p}$ is the radius of the SPION; $\vec{v}$ is the particle drift velocity; and the magnetic moment is $\vec{m}=\frac{4}{3}\pi R_{p}^{3}\vec{M}$ (assuming uniformly magnetized spherical SPIONs), where $\vec{M}$ is the magnetization. The force balance can be rewritten as follows:

(8) $$F=\frac{4}{3}\pi R_{p}^{3}\nabla \left(\vec{M}\cdot \vec{B}\right)-6\mu \eta R_{p}\vec{v}$$

Assuming that the SPION rapidly reaches its terminal velocity, its drift velocity during a magnetic pulse is equal to

(9) $$\vec{v}=\frac{2R_{p}^{2}}{9\eta }\nabla \left(\vec{M}\cdot \vec{B}\right)$$

Substituting this result into the equation of continuity in Equation (4), it can be rewritten as

(10) $$\frac{\partial c}{\partial t}=\frac{kT}{6\pi \eta R_{p}}\nabla^{2}c-\nabla \cdot \left(\left(\frac{2R_{p}^{2}}{9\eta }\nabla \left(M\cdot B\right)\right)c\right)$$

The dependency of β on particle size, from Equations (6), (9), and (10), can then be expressed as shown in Equation (11).

(11) $$\beta =\frac{g_{\left(x\right)}}{R_{p}}+h_{\left(x\right)}R_{P}^{2}$$

where $g_{\left(x\right)}=-\frac{kT}{6\pi \eta }\nabla^{2}c$ and $h_{\left(x\right)}=\frac{2}{9\eta }\left(\nabla \left(M\cdot B\right)\cdot \nabla c+c\nabla^{2}\left(M\cdot B\right)\right)$. This equation, with the function $g_{\left(x\right)}$ capturing the effects of diffusion and $h_{\left(x\right)}$ capturing the result of magnetic attraction, was used to model the change in concentration ($\frac{\partial c}{\partial t}$) of SPIONs of various sizes during the period of applied magnetic field (i.e., during the ON phase of DMF).

**Figure A1:**
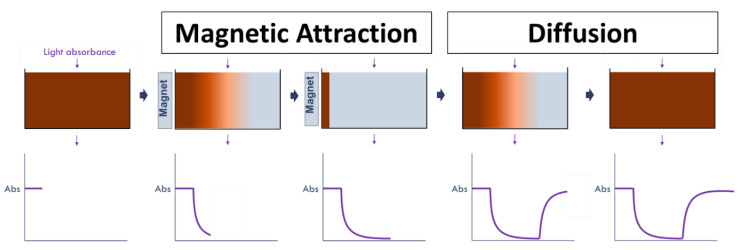
The experimental setup used to simulate the different particle mobility characteristics of SPIONS of different sizes during the magnetic attraction and diffusion steps of PMF: A solution of SPIONs was placed into a 96-well plate, and the absorbance was recorded at a wavelength of 450 nm over time at the center point of the well. During the magnetic attraction portion of the study, a magnet was introduced adjacent to the well, and particles were observed to migrate towards the magnet, as observed by a decrease in the absorbance reading at the center point of the well over time. For the diffusion study, the magnet was removed after SPIONs were magnetically concentrated on one side, and SPION concentration was observed to increase over time at the center of the well.

**Figure A2:**
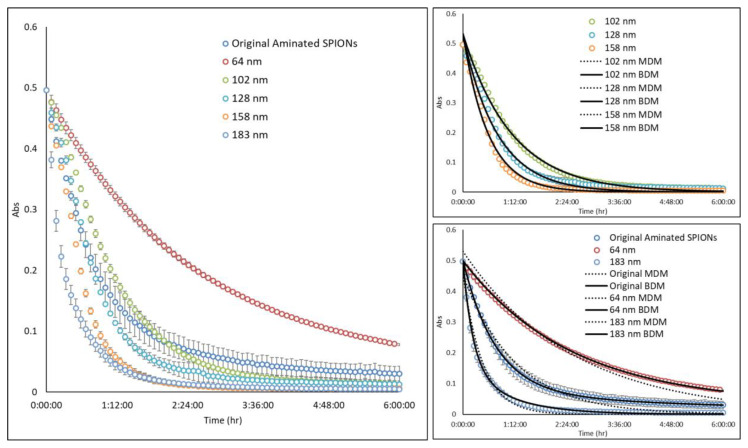
Magnetic attraction study: Multicore aminated SPIONs (ø 137 nm; PdI = 0.18) were fractionated by DMF into 5 subpopulations having average diameters of 64, 102, 128, 158, and 183 nm and PdIs of 0.09, 0.06, 0.08, 0.08, 0.08, and 0.1, respectively. Each of the samples was placed into a 96-well plate, and the absorbance at wavelength of 450 nm was measured with time at a fixed, center point within the well. When a magnet was introduced adjacent to the well, particles were observed to migrate towards the magnet and the SPION concentration at the center decreased with time. The rate at which SPIONs migrated towards the magnet was dependent on particle size. All experimental results were fitted by a mono-exponential decay model (MDM) and bi-exponential decay model (BDM).

Since the β parameter is a function of SPION size, its magnitude was experimentally determined for SPION particles of varying diameters. For example, aminated SPIONs with an average diameter of 137 nm and PdI of 0.18 were separated by DMF into five fractions having average diameters of 64, 102, 128, 158, and 183 nm and PdIs of 0.09, 0.06, 0.08, 0.08, and 0.1, respectively. Next, as illustrated in Figure A1, a solution of each fractionated SPION was placed into a 96-well plate and the absorbance at wavelength of 450 nm was measured with time at a fixed, center point within the well. When a magnet was introduced adjacent to the well, SPION concentration at the center decreased with time, as shown in Figure A2, due to particle migration towards the magnet. Interestingly, the data for the middle size fractions (i.e., 102, 128, and 158 nm SPIONs) were well described by a mono-exponential decay model, similar to Equation (5). Data for the original 137-nm SPIONs and the 64- and 183-nm SPIONs, all of which had PdIs of 0.09 or greater, on the other hand, were better fit with a bi-exponential model, reflecting the inhomogeneity of these three samples. Consistent with expectations, the original aminated SPIONs showed the greatest PdI, and the largest and the smallest fractions had broader particle size distributions since they included all particles above or below, respectively, their average size. This finding demonstrates the potential of the DMF method to produce MNP having a more homogeneous property distribution.

**Figure 4:**
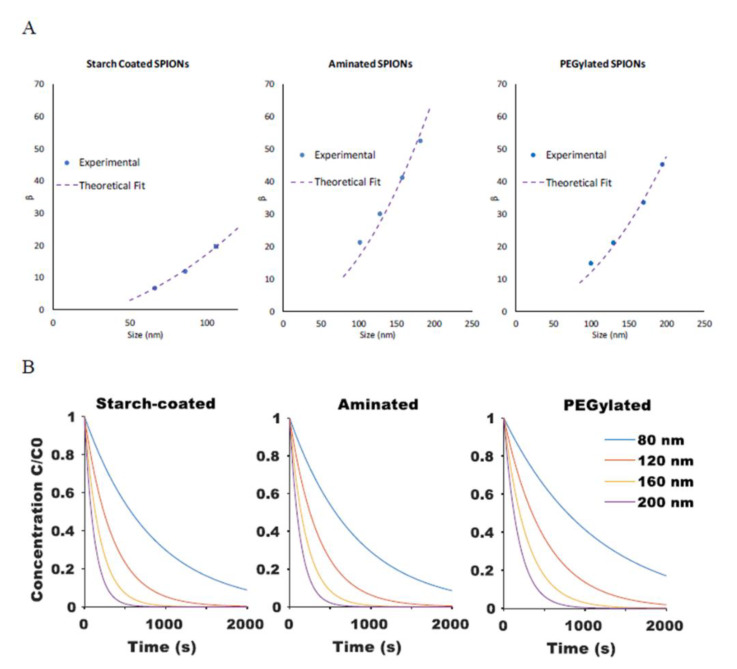
(**A**) Comparison of the magnetic attraction rate constant *β* between experimental results (extracted from mono-exponential fit of experimental magnetic attraction data) and theoretical fit from Equation (12) for starch-coated, aminated, and PEG-modified SPIONs. (**B**) Model-generated curves for change in relative SPION concentration at a fixed point based on Equations (5) and (12) when exposed to a magnetic field.

The experimental β values for each type of SPION was extracted from data sets that could be fit with the mono-exponential decay model (i.e., by fitting Equation (5)). Additionally, theoretical β values for particles of different sizes were calculated using Equation (11), simplified for a fixed position as Equation (12),

(12) $$\beta =\frac{A}{R_{p}}-B\;R_{p}^{2}$$

where *A* and *B* are fitting parameters that account for g(x) and h(x). Model-predicted theoretical β values agreed well with the experimental results for SPIONs of different sizes and for SPIONs with different surface coatings (*R*^2^ values of 1, 0.95, and 0.98 for starch-coated, aminated, and PEGylated SPIONs, respectively), as shown in Figure 4A. The model was then used to predict the rate of SPION movement for particles of varying size and surface coatings within a given magnetic field, as shown in Figure 4B.

#### 2.3.3. Simulation of SPION Diffusion in the Absence of an Applied Magnetic Field

During the OFF cycle of DMF, Brownian motion induces the free diffusion of SPIONs away from the site of magnetic accumulation, with smaller SPIONs having a faster diffusion rate than large SPIONs as described by the Stokes–Einstein equation. Assuming negligible bulk flow conditions, Equation (4) simplifies to Fick’s Second Law:

(13) $$\frac{\partial C}{\partial t}=D\frac{\partial^{2}C}{\partial x^{2}}$$

Equation (14) shows the time-dependent solution of this differential equation,

(14) $$C=C_{0}\left(1-e^{-\gamma \cdot D\cdot t_{d}}\right)=C_{0}\left(1-e^{-\alpha \cdot t_{d}}\right)$$

where α is a fitting constant, *D* is the diffusivity coefficient, *γ* is an integration constant, and $t_{d}$ is the time duration for diffusion (i.e., the duration of the OFF phase).

**Figure 5:**
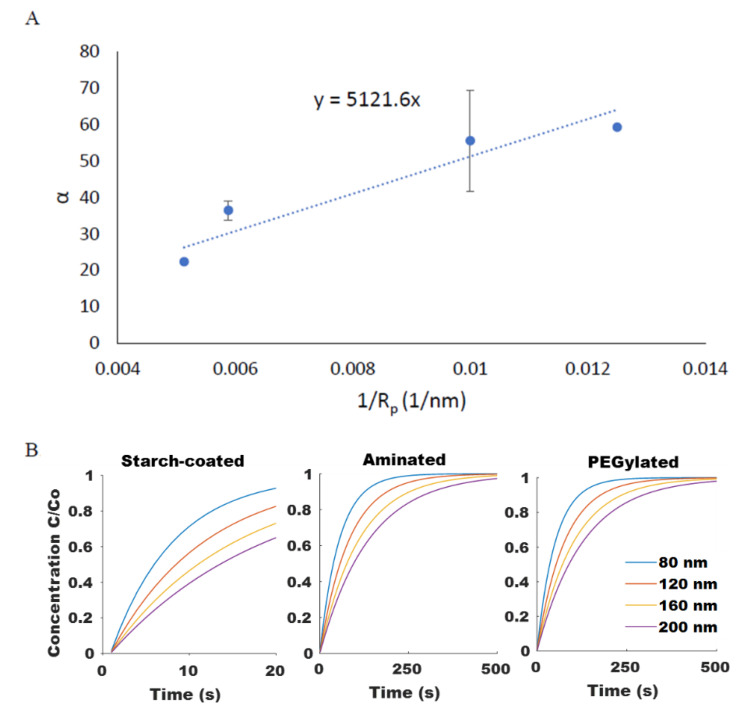
(**A**) The size dependency of SPION diffusion rate, α, shown for PEGylated SPIONs agreed well (*R*^2^ = 0.89) with the Stokes–Einstein equation. (**B**) Computationally modeled diffusion profiles for SPIONs of varying size and surface chemistry were generated.

Experimental measurements of the rate of particle diffusion of SPIONs were obtained using the same well-plate setup shown in Figure A1. In this instance, however, particle concentration measurements were taken after the SPIONs were magnetically concentrated on one side and the applied magnetic field was then removed. The time-dependent concentration at a fixed point was fitted with Equation (14) for each of the different SPION formulations to obtain values for α; results are shown in Figure 5A. These parameters could then be applied to predict the rate of SPION diffusion for particles of varying size and surface coatings, as shown in Figure 5B.

#### 2.3.4. Optimization of the DMF Method

**Figure 6:**
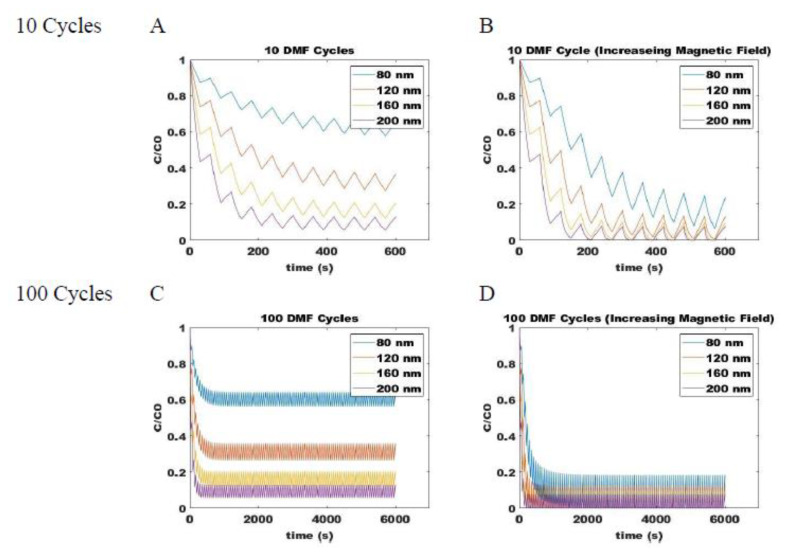
Predicted concentrations of various sized SPIONs during DMF process with a constant magnetic field through (**A**) 10 cycles and (**C**) 100 cycles and with increasing magnetic field strengths through (**B**) 10 cycles and (**D**) 100 cycles. Shown for different size SPIONs: blue is 80 nm, orange is 120 nm, yellow is 160 nm, and purple is 200 nm.

Combining the ON (Equation (5)) and OFF (Equation (14)) models for magnetic attraction and free diffusion of SPIONs, respectively, allows for determination of the dynamic changes in particle distribution within the system. Process parameters that can be varied during a pulse sequence include the duration of applied field (*t_m_*), the duration during which diffusion occurs when the field is removed (*t_d_*), the magnitude of the applied magnetic field, and the number of repeat cycles (*n*). For example, the dynamic change in concentration of 80-, 120-, 160-, and 200-nm SPIONs at a fixed position are shown with up to 100-cycle DMF with a fixed magnetic field strength (Figure 6A,C) and with cycles of increasing field strength (Figure 6B,D). The *y*-axis on the plot shows the fraction of SPIONs of a given size relative to its own original concentration at that position. All particles, therefore, start at an initial concentration fraction of one, and a decrease indicates its depletion due to accumulation at a position closer to the applied field source. Particles that show a more rapid decrease in concentration fraction can be expected to more rapidly sediment at the underlying surface. When pulses of a fixed magnetic field strength are applied, the larger particles are observed to move more rapidly along the field gradient than smaller particles. However, even with 100-cycles, an equilibrium concentration range is achieved at conditions where the magnetic and diffusive effects are balanced. When the field strength is increased between pulses, however, the imbalance is maintained, and particles will continue to migrate towards the applied magnetic field, allowing for stratification of SPIONs according to size on the surface of magnetic beads.

During a DMF cycle, larger SPIONs experience a faster magnetic attraction rate (during the ON phase) and a slower diffusion rate (away during the OFF phase) compared to smaller SPIONs. Therefore, a unique time required for capture of 95% of particles of a given size, $R_{p}$, (i.e., time for concentration to decrease to 0.05 of the original in Figure 4B) can be defined for each SPION and is denoted as $t_{c}\left(x,\;R_{p}\right)$. Additionally, an elapsed time, $t\left(c,\;R_{p}\right)$, for the system can be defined for each particle size based on the current concentration fraction (*c*). Then, for a given position, *x*, the time required, $t_{r}$, for the concentration of a particle of a specific size to reach 95% capture by the iron bead is calculated as

(15) $$t_{r}\left(c,\;R_{P}\right)=t_{c}\left(x,\;R_{p}\right)-t\left(c,\;R_{p}\right).$$

**Figure 7:**
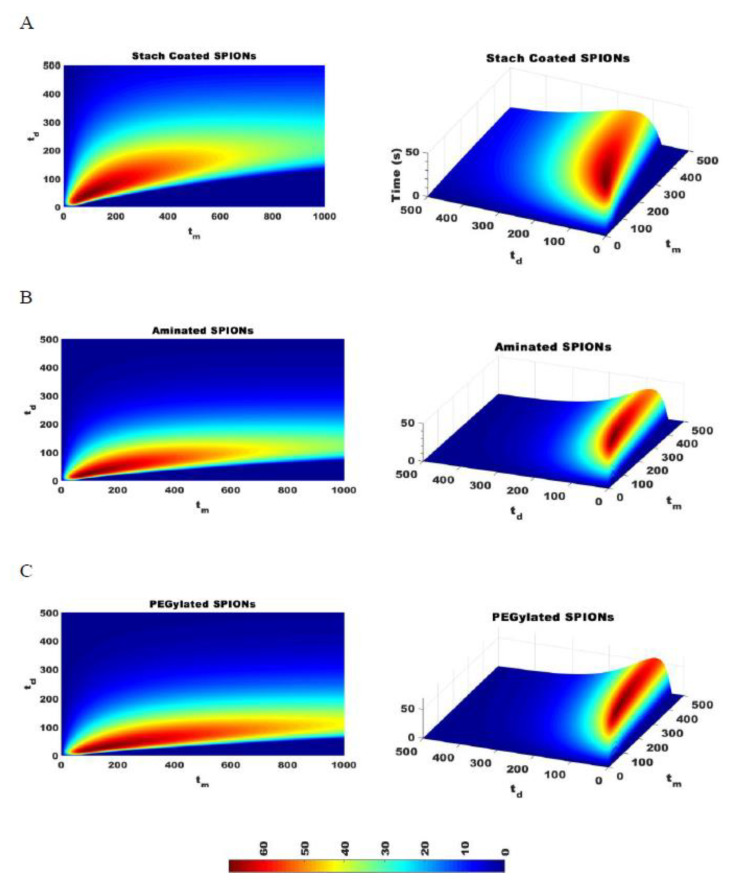
The difference in time required to attract 95% of SPIONs of two different sizes, $\left|∆t_{r}\right|$, is calculated for SPIONs of 70 and 140 nm with a constant magnetic strength DMF. The optimal pulse sequence for starch coated SPIONs was $t_{m}$ = 70 and $t_{d}$ = 35 (**A**), the optimal pulse sequence for aminated SPIONs is $t_{m}$ = 80 and $t_{d}$ = 20 (**B**), and the optimal pulse sequence for PEGylated SPIONs is $t_{m}$ = 110 and $t_{d}$ = 20 (**C**).

**Figure 8:**
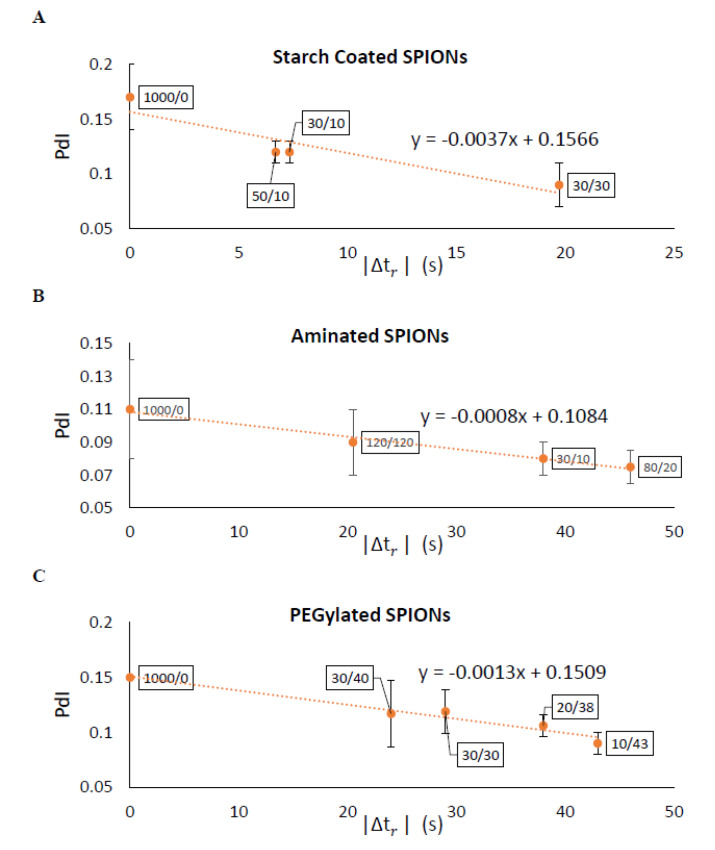
Experimental results verified the model predications that a larger |∆*t_r_*| would yield narrower particle size distributions (i.e., smaller PdI) with DMF. The data labels are the magnetic pulse sequences used for each separation. Measured PdIs showed strong agreement with the model for (**A**) starch–coated, (**B**) aminated, and (**C**) PEGlyated SPIONs.

The effectiveness of separating two different sized particles is then determined as $\left|∆t_{r}\right|$, which is the difference in $t_{r}$ of the two particles and which depends on the magnetic attraction and diffusion rates. As $\left|∆t_{r}\right|$ increases, the separation of particles improves. Figure 7 shows $\left|∆t_{r}\right|$ for pulses with varying durations of magnetic attraction, $t_{m}$, and particle diffusion, $t_{d}$, for SPIONs of 70 and 140 nm average diameters. The optimal pulse sequence was found to be dependent on particle type, with $t_{m}$= 70 s and $t_{d}$ = 35 s for starch-coated SPIONs, with $t_{m}$= 80 s and $t_{d}$ = 20 s for aminated SPIONs, and with $t_{m}$= 110 s and $t_{d}$ = 20 s for PEGylated SPIONs. Various pulse sequences that generate different $\left|∆t_{r}\right|$ values were then used to fractionate SPIONs, and the average PdI values for all the fractions were compared. As expected, the average PdI values decreased as $\left|∆t_{r}\right|$ was increased for all three SPION formulations (see Figure 8). This suggested that this simple model could be used to determine the optimal pulse sequence for SPIONs of different formulations based on simple magnetic attraction and diffusion experimental data. Looking more closely at Figure 8A, it will be noted that two of the points tested have very similar $\left|∆t_{r}\right|$ values of 6.7 and 7.33 s. While these two points are closely placed on this plot, they are obtained with very different magnetic pulse sequences—demonstrating the robustness of this method in optimizing separation of SPIONs and the utility of the $\left|∆t_{r}\right|$ parameter.

It should again be noted the several simplifying assumptions (e.g., magnetic core structure and particle size) we made in the analysis. While this did not appear to impact our ability to tune the DMF process, a more detailed model could potentially lead to additional insight that enables further fine tuning. On the other hand, while results presented here were specific to SPIONs of a particular size, the DMF method could be extended to other MNPs as well with the only limitation being that they respond to an applied magnetic field gradient and diffuse away in the absence of a magnetic field. For example, the DMF method could possibly be applied to MNPs showing remnant magnetization (i.e., magnetization hysteresis) if the thermal energy of the system is sufficient to overcome any remaining interparticle interactions. The system temperature could be raised as one means to achieve this effect. Additionally, solvent parameters could also be tuned to modulate particle diffusion and interactions under the applied magnetic field. Also, while we have demonstrated the DMF method using MNP and applied magnetic fields, a similar approach could certainly be considered for other systems that allow for pulsed application of field gradients (e.g., charged particles and an applied electrical field). Therefore, the exciting results presented in this work specifically for separation of SPIONs could lead to other avenues of pursuit to improve homogeneity of nanoparticle formulations.

## 3. Materials and Methods

### 3.1. Synthesis of Aminated and PEGylated SPIONs

SPIONs (FluidMAG-D; Chemicell, Germany) used in this study were measured by a superconducting quantum interference device (SQUID) to have a magnetic saturation of 27 emu/g. The surface of the starch-coated SPIONs was crosslinked and further aminated according to a previously published method [43]. Briefly, 2 mL of starch-coated SPIONs (25 mg Fe/mL) was incubated with 2.6 mL of 6 M NaOH for 15 min. Next, 1.3 mL of epichlorohydrin was added and incubated for 24 h at room temperature. The crosslinked SPIONs were dialyzed against DI water for 24 h (Float-A-Lyzer G2, 8-10 kDa MW; Spectrum Laboratories, Rancho Dominguez, CA, USA) to remove excess reagents and then incubated with 2 mL ammonium hydroxide (28%–30% ammonia) at room temperature for 24 h. The aminated SPIONs were dialyzed against DI water for 48 h (Float-A-Lyzer G2, 8–10 kDa MW; Spectrum Laboratories, CA, USA). Meanwhile, PEGylated SPIONs were generated by first dissolving 15 mg mPEG-NHS (Nanocs; New York, NY, USA) in 300 µL dimethyl sulfoxide, 300 µL deionized water, and 300 µL phosphate buffer and then by mixing with 300 µL of aminated SPIONs. The final solution was placed on a shaker for 24 h at room temperature. The resulting PEGylated SPIONs were purified using a MagneSphere Technology magnetic separation stand (Promega, Madison, WI, USA).

### 3.2. Characterization of SPIONs

#### 3.2.1. Size, Polydispersity Index, and Zeta Potential

ZetaSizer Nano ZS90 (Malvern, UK) was used to measure particle hydrodynamic diameters (Z-average size), particle size distribution (PI), and zeta potentials. All measurements were taken in triplicates.

#### 3.2.2. Iron Quantification Assay

The iron content of SPIONs was determined using a quantitative ferrozine assay. A KMnO_4_/HCl solution was made by mixing equal volumes of 4.5% *w*/*v* KMnO_4_ with 1.4 M HCl. Briefly, 200 μL of the SPION sample was added to 230 μL of the KMnO_4_/HCl solution and incubated for 2 h at 60 °C followed by 10-min cooling; 170 μL of the mixture was transferred to a 96-well plate, and 30 µL of ferrozine solution (6.5 mM ferrozine, 6.5 mM Neocuproine, 2.5 M ammonium acetate, and 1 M ascorbic acid in H_2_O) was added. The mixture was incubated at room temperature for 30 min. The absorbance of the samples was measured at 550 nm using a SpectraMax i3 plate reader (Molecular Devices, Sunnyvale, CA, USA). Standard curves (*R*^2^ = 0.9999) were created using eight known concentrations between 7.5 and 250 µg Fe/mL of an iron standard solution.

### 3.3. Setup for Magnetic Fractionation

An iron bead packed LS Column (Miltenyi Biotec, Sunnyvale, CA, USA) was used for separation of SPIONs by MFFF and DMF. The column was placed within the cavity of a 22-W electric coil, which was connected to a DC power supply (Hewlett Packard 6543A), to provide an external magnetic field. Magnetic field strength was modulated by adjusting the current passing through the coil. Due to constraints of available equipment, the maximum applied magnetic field was limited to 24 mT and test fields for this study were selected between 0–24 mT.

#### 3.3.1. Magnetic Field Flow Fractionation (MFFF)

MFFF was performed according to literature with minor alterations [44]. The magnetic column was set within the coil with a constant magnetic field of 24 mT. An aqueous suspension of SPION was allowed to flow through the magnetic column by gravity. Fluid was collected at column exit and added back to the top of column repeatedly until the fluid turned clear (indicating that SPIONs were fully captured by the column). A continuous water flow of 1.5 mL/min was then introduced by a peristaltic pump, and the external magnetic field strength was decreased in stepwise manner. Released SPIONs were captured by a second magnetic column magnetized by a 220-mT bar magnet. Collection of each fractionation required approximately 8 min.

#### 3.3.2. Diffusive Magnetic Fractionation (DMF-0 and DMF-9)

For DMF-0, SPION solution was first introduced into a magnetic column with the column outlet blocked to prevent flow. A constant magnetic field of 24 mT was then applied for 8 min. Next, a continuous water flow of 1.5 mL/min was introduced by a peristaltic pump while the external magnetic field strength was maintained at 24 mT for another 8 min. Following this, the external magnetic field strength was decreased in a stepwise manner and any released SPIONs captured by a second magnetic column were magnetized by a 220-mT bar magnet. Collection of each fraction required 5–8 min.

For DMF-9, nine cycles of pulsed magnetic field were applied to capture SPIONs on the surface of the magnetic beads, with 70 s of applied magnetic field (ON) and 35 s without magnetic field (OFF cycle). In this case, the magnetic field strength was increased gradually after each cycle from 0.4 to 0.8, 1.6, 4, 8, 12, 16, 22, and finally 24 mT. After reaching the maximum field strength of 24 mT, the field was maintained ON for 8 min before a continuous water flow of 1.5 mL/min was introduced by a peristaltic pump for another 8 min. Afterwards, the external magnetic field strength was decreased in a stepwise manner and any released SPIONs captured by a second magnetic column magnetized by a 220-mT bar magnet. Each fractionation required 5–8 min to collect.

#### 3.3.3. Versatility and Scalability Study

Four iron bead packed LS Columns (Miltenyi Biotec, Sunnyvale, CA, USA) were used with each column having a portion of its iron beads removed, including 0%, 25%, 50%, and 75% removal, to yield varying capacities of 2, 1.5, 1.0, and 0.5 mg of SPIONs, respectively. The separation process followed a general DMF-9 setting and generated four fractions of SPIONs, which were obtained with magnetic field strengths of 24, 16, 8, and 0 mT

### 3.4. Determination of Rate of SPION Capture by Magnetic Attraction

A 250-mT bar magnetic was placed inside a 96-well plate with its magnetic pole pointing towards an adjacent well. An aqueous SPION suspension was then added into the adjacent well and absorbance measured at 450 nm (SpectraMax i3, Molecular Device, San Jose, CA, USA) to determine SPION concentration at the center of the well. Absorbance measurements were taken every 10 min for 6 h.

### 3.5. Determination of Rate of SPION Free Diffusion in the Absence of a Magnetic Field

Following the magnetic attraction study, the 250-mT bar magnet was removed from the 96-well plate and the rate of SPION diffusion was determined. Absorbance measurements were taken at the center of the well every 10 s for several minutes or at multiple positions inside the well every minute for 20 min.

## 4. Conclusions

A major challenge to broader adaption of multicore MNP-based biomedical technologies is related to their poor particle size distribution, which results in suboptimal performance, variable biodistribution and blood clearance pharmacokinetics, multistage clearance, and varied MRI contrast properties. Producing monodisperse SPIONs with well-defined physiochemical properties is necessary to ensure consistent results in biomedical application and to enable successful clinical translation. The goal of this study was not to discover a new formulation but to develop a post-synthesis size separation method that enables the development of more homogenous formulations. DMF was developed as an improvement to the conventional MFFF method for size-selective separation of polydisperse MNPs. DMF demonstrated significantly improved separation quality and control over particle size distribution compared to the conventional MFFF method. This is due to the use of a pulsed magnetic field, which exploits differences in both the diffusivity and the magnetic force experienced by SPIONs of different sizes. DMF was able to produce SPIONs with excellent polydispersity index and a 95% recovery of SPIONs. Mathematical models were developed to predict the average size of SPIONs produced with each fractionation and to optimize the pulse sequence of the pulsed magnetic field. We believe that this research provides a tool that will allow researchers to better understand the relationship between the physical properties of MNPs and their biological properties.

## 5. Patents

This work has resulted in U.S. Utility Patent application serial no. 16/040,096.

## Abbreviations

DMF	diffusive magnetic fractionation
DMF-0	DMF with zero PMF cycles
DMF-9	DMF with nine PMF cycles
MFFF	magnetic field flow fractionation
MNPs	Magnetic nanoparticles
MRI	magnetic resonance imaging
PdI	Polydispersity Index
PEG	polyethylene glycol
PMF	pulsed magnetic field
SEC	size exclusion chromatography
SPION	superparamagnetic iron oxide nanoparticles
